# The two common polymorphic forms of human NRH-quinone oxidoreductase 2 (NQO2) have different biochemical properties

**DOI:** 10.1016/j.febslet.2014.02.063

**Published:** 2014-05-02

**Authors:** Clare F. Megarity, James R.E. Gill, M. Clare Caraher, Ian J. Stratford, Karen A. Nolan, David J. Timson

**Affiliations:** a School of Biological Sciences, Queen’s University Belfast, Medical Biology Centre, 97 Lisburn Road, Belfast BT9 7BL, UK; b Manchester Pharmacy School, The University of Manchester, Stopford Building, Oxford Road, Manchester M13 9PT, UK

**Keywords:** rs1143684, Resveratrol, Cooperativity, Dihydronicotinamide riboside

## Abstract

There are two common forms of NRH-quinone oxidoreductase 2 (NQO2) in the human population resulting from SNP rs1143684. One has phenylalanine at position 47 (NQO2-F47) and the other leucine (NQO2-L47). Using recombinant proteins, we show that these variants have similar steady state kinetic parameters, although NQO2-L47 has a slightly lower specificity constant. NQO2-L47 is less stable towards proteolytic digestion and thermal denaturation than NQO2-F47. Both forms are inhibited by resveratrol, but NQO2-F47 shows negative cooperativity with this inhibitor. Thus these data demonstrate, for the first time, clear biochemical differences between the variants which help explain previous biomedical and epidemiological findings.

## Highlights

• SNP rs1143684 results in either a Phe or Leu at position 47 in human NQO2.
• NQO2-L47 has a slightly lower *k*_cat_/*K*_m_; it also has a lower *K*_i,app_ with resveratrol.
• NQO2-L47 is more unstable to proteolysis and thermal denaturation.
• NQO2-F47 (but not NQO2-L47) shows negative cooperativity towards resveratrol.
• NQO2-L47 exists as multiple conformers in solution.

## 1 Introduction

There are two members of the quinone oxidoreductase family in humans. Of these, NAD(P)H quinone oxidoreductase 1 (NQO1, DT-diaphorase, EC 1.6.5.2) is the better characterised of the two [1,2]. This enzyme is believed to be involved in vitamin K metabolism and in reducing the cellular quinone concentration, thus preventing build-up of reactive oxygen species [3–5]. It also binds to, and stabilises, the apoptosis regulator p53 [6–11]. Its up-regulation in some cancer cells and its role in the conversion of some pro-drugs (e.g. mitomycin c) to their pharmacologically active forms has resulted in considerable interest in targeting this enzyme for the development of novel cancer chemotherapies [5,12–16]. Much less is known about NRH quinone oxidoreductase 2 (NQO2, EC 1.10.99.2). Like NQO1, it has a tightly bound FAD cofactor which is reduced as part of the enzyme’s catalytic cycle [17]. However, the reductant differs between the two enzymes. While NQO1 can utilise both NADH and NADPH with almost equal efficiency [18], NQO2 has only low levels of activity with these compounds [19,20]. In vitro experiments have demonstrated that the non-physiological compound dihydronicotinamide riboside (NRH) acts as a good substrate [19]. In addition, N-methyldihydronicotinamide and dihydrobenzylnicotamide, have been reported to be able to act as reductants in vitro and 1-carbamoylmethyl-1,4-dihydronicotinamide (caricotamide, EP 0152R) is a cell-permeable NRH analogue which enables the reductive activation of the pro-drug CB1954 (5-(aziridin-1-yl)-2,4-dinitrobenzamide) by NQO2 [21–26].

Both NQO1 and NQO2 have substituted enzyme (ping-pong) mechanisms in which, following initial reduction of the FAD molecule by NAD(P)H or NRH, the first product leaves the active site and is replaced by the second substrate which then undergoes a two electron reduction, regenerating FAD [17,27]. The active sites can accommodate and reduce a wide variety of molecules including quinones, nitro-compounds and iron (III) ions [28–32]. NQO1 is inhibited by the anticoagulant dicoumarol and many structurally related compounds [33]. In contrast, NQO2 is only weakly inhibited by dicoumarol, but is more strongly competitively inhibited by resveratrol [34]. Ingestion of this compound, which is present in red wine, peanuts, mulberry fruits and dark chocolate, has recently been linked to increased longevity in some species including *Saccharomyces cerevisiae* and *Drosophila melanogaster* although the effect in humans remains controversial [35–41].

Interestingly, there are two common forms of the *NQO2* gene in the human population. These differ at codon 47 (SNP rs1143684), which can either be CTT (encoding leucine) or TTT (phenylalanine). Thus, there are two corresponding forms of the enzyme, one with phenylalanine at position 47 (NQO2-F47) and the other with leucine (NQO2-L47). Residue 47 is not part of the enzyme’s active site but is close to the dimer interface [42]. Of these NQO2-F47 is the more common in the human population. Estimates of the allele frequency for CTT (Leu) vary from 2% in African populations to 33% in East Asians; the estimated frequency in Europeans is 20% (http://e72.ensembl.org/Homo_sapiens/Variation/Population?align=548;db=core;r=6:3009890-3010890;v=rs1143684;vdb=variation;vf=907401) [43]. NQO2-L47 has been associated with more rapid decline in memory recall and with better prognosis in breast cancer [44–46]. However, not all studies have demonstrated statistically significant associations between this polymorphism and cancer prognosis [47]. NQO2-L47 has been reported to have lower activity than NQO2-F47 in cell extracts [48] but, to date, little other biochemical data has been collected on the two variants. Here, we compared the biochemical properties of the two forms of NQO2 and provide a molecular explanation for the reduced cellular activity of NQO2-L47.

## 2 Materials and methods

### 2.1 Expression and purification of human NQO2

The coding sequence for human NQO2 was amplified by PCR from a plasmid kindly supplied by Dr. David Jamieson (University of Newcastle-upon-Tyne, UK) using primers which enabled the insertion of the amplicon into pET46-Ek-LIC (Merck, Nottingham, UK) according to the manufacturer’s instructions. This sequence encodes a phenylalanine at codon 47 and site directed mutagenesis using the QuickChange protocol [49] was used to change codon 47 to one that encodes leucine. In both cases the entire coding sequence was verified by DNA sequencing (GATC, London, UK). The vector inserts sequence coding for the residues MAHHHHHHVDDDDK at the 5′-end of the gene and so enables purification of the recombinant proteins by nickel affinity chromatography (His-Select, Sigma, Poole, UK) using the same method as employed for other proteins in our laboratory, e.g. [50]. Protein concentrations were estimated by the method of Bradford using BSA as a standard [51].

### 2.2 NQO2 activity assays

NQO2 activity was measured at 37 °C in 50 mM HEPES, pH 7.3 using NRH as the electron donor and DCPIP as the electron acceptor. NRH was synthesised from NADH [52]. NADH (0.50 g, 0.69 mmol) was dissolved in 20.0 ml of 0.4 M sodium carbonate/bicarbonate buffer, pH 10.0, and incubated at 37 °C for 16 h with 0.1 unit of phosphodiesterase 1 type IV and 500 units of alkaline phosphatase type VII-S. After complete digestion of NADH, the mixture was freeze dried. The dried powder was extracted with methanol (five times 6 ml), and this methanol extract was dried by rotary evaporation and dissolved in 5.0 ml of water. The NRH was then purified in 1 ml batches by preparative HPLC on a microsorb C18 column (21.2 by 250 mm), eluted with 10% methanol in water over 15 min at a flow rate of 15.0 ml min^−^^1^. The NRH peak was detected absorption at 350 nm. This peak from each injection was collected, freeze-dried and stored at 4 °C.

All NQO2 activity assays were carried out in triplicate (same enzyme preparation, same enzyme dilution) in the same 96-well plate with readings taken every 5 s and initial, linear rates of change in absorbance at 600 nm measured. Over the NRH concentration range studied, the linear portion of the progress curve lasted at least 50 s. To ensure that initial rates were measured, reactions were done in batches of four wells at a time. Each reaction rate was corrected by subtraction of the background rate of DCPIP reduction by NRH estimated experimentally in a parallel reaction containing the same components except enzyme. This background rate never exceeded 10% of the total rate and the estimated pseudo-first order rate constant for the non-enzymatic reduction under these conditions was (6.7 ± 0.4) × 10^−^^5^ s^−^^1^. The enzyme-catalyzed rate was divided by the dimeric enzyme concentration (2.5 nM for both variants). The apparent Michaelis constant (*K*_m,app_) and apparent turnover number (*k*_cat,app_) values were determined by plotting enzyme-catalyzed rate (*v*) divided by enzyme concentration ([E]) against the corresponding NRH concentration. The data were fitted to Eq. (1) using non-linear curve fitting in GraphPad Prism 6 (GraphPad Software Inc., CA, USA).

(1) $$\nu /[\text{E}]=k_{\text{cat,app}}[\text{NRH}]/K_{\text{m,app}}+[\text{NRH}]$$

Linearized Hill plots were constructed to determine the Hill coefficient (*h*) according to Eq. (2).

(2) $$-\log_{10}((\nu /[\text{E}])/(k_{\text{cat,app}}-(\nu /[\text{E}])))=-h.\log_{10}[\text{NRH}]-\log_{10}k_{0.5\text{,app}}$$

### 2.3 Inhibition by resveratrol

The effect of resveratrol (0–640 nM; initially dissolved in 100% DMSO and diluted such that the final volume of DMSO in the assay was 0.5% v/v) on the enzyme-catalysed rate was measured at two concentrations of NRH (50 and 100 μM) with a constant DCPIP concentration (70 μM). Dixon plots were constructed to obtain the apparent inhibition constant, *K*_i,app_. The degree of cooperativity towards resveratrol was determined by fitting the data obtained using 50 μM NRH and 70 μM DCPIP to Eq. (3), which follows from obtaining the algebraic ratio of the steady state rate equations in the absence of (*v*_0_), and the presence of (*v*), the competitive inhibitor resveratrol and collecting the constant terms – i.e. *K*_m_, [NRH], [DCPIP], *K*_ic_ (the competitive inhibition constant) – into a single term, *Z*.

(3) $$1-(\nu /\nu_{0})={\left[\text{resveratrol}\right]}^{h}/(Z^{h}+{\left[\text{resveratrol}\right]}^{h})$$

For both variants, the fit to this equation and a similar one lacking the Hill coefficient (*h*) were compared using an *F*-test (implemented in GraphPad Prism) and results from the equation judged to be a better fit reported. Linearized Hill plots were also constructed using Eq. (4) using the same data for display purposes.

(4) $$-\log_{10}(\nu /(\nu_{0}-\nu ))=-h.\log_{10}[\text{resveratrol}]-\log_{10}Z$$

### 2.4 Limited proteolysis, crosslinking and determination of flavin content

Limited proteolysis with trypsin, chymotrypsin and subtilisin was carried out as previously described and analysed using tris-tricine SDS–PAGE [53,54]. Crosslinking with *bis*sulfosuccinimidylsuberate (BS^3^) and *N*-(3-Dimethylaminopropyl)-*N*′-ethylcarbodiimide hydrochloride (EDC) was performed as previously described and analysed by tris-glycine SDS–PAGE [53].

The flavin content of the recombinant NQO2 variants was determined by first obtaining an absorption spectrum of the proteins from 250 to 550 nm (Cary 100 Scan). From the absorption at 375 nm and 450 nm, the contribution of the flavin to absorption at 280 nm was calculated. The remaining absorbance at this wavelength was assumed to be due to protein and used to calculate the protein concentration using *ε*_280nm_ = 44,920 l mol^−^^1^ cm^−^^1^ (estimated using ProtParam in Expasy [55]). An aliquot (500 μl) of protein was then heated at 95 °C for 4 min to release the FAD followed by centrifugation at 14 000×*g* for 1 min to remove the precipitated protein [56]. The supernatant was removed and the volume was restored to 500 μl to correct for losses resulting from heating. The absorbance at 375 and 450 nm was determined and used to estimate the concentration of flavin. The occupancy per monomer was calculated as (flavin concentration/NQO2 active site concentration) × 100%.

### 2.5 Thermal scanning fluorimetry (TSF)

Each variant (0.5 μM in 50 mM HEPES-OH, pH 7.5) was subjected to an increase in temperature from 25 to 95 °C (increments of 1 K and remaining at each temperature for 5 s) in a Rotor-Gene Q cycler (Qiagen) (high resolution melt protocol, no gain optimisation; excitation at 460 nm and emission at 510 nm) exploiting the fluorescence of the cofactor, FAD, which increases when it is released from the enzyme into solution [57]. The melting temperature (*T*_m_) was determined from the first derivative of the melting curve, using the inbuilt analysis software. Stock solutions of resveratrol (in 100% DMSO) and dicumarol (in 0.13 M NaOH) were prepared such that, when diluted into the assay solution, the final concentration of solvent was 0.5% (v/v). Nicotinamide was dissolved in 50 mM HEPES-OH, pH 7.5. The change in melting temperature (Δ*T*_m_) at each concentration of ligand was determined and data fitted to Eq. (5).

(5) $$\Delta T_{\text{m}}=\Delta T_{\text{m,max}}[\text{ligand}]/(K_{\text{D,app}}+[\text{ligand}])$$

where Δ*T*_m,__max_ is the maximum, limiting change in *T*_m_ and *K*_D,app_ is the apparent dissociation constant.

## 3 Results and discussion

### 3.1 Expression and characterisation of recombinant NQO2-F47 and NQO2-L47

Both variants of NQO2 could be expressed in, and purified from, *Escherichia* *coli* (Supplementary Fig. S1). The flavin cofactor of the recombinant NQO2 variants was released by thermal denaturation of the proteins. The absorption spectrum maxima were consistent with those expected for a mixture of FAD and FMN (well defined peaks at 266 and 375 nm and a broad peak 446–450 nm) [58]. This is in contrast to some previous reports where only FMN or only FAD has been detected as the flavin cofactor present in recombinant NQO2 [19,56]. It is not expected to affect the enzyme activity greatly since the standard redox potentials of both compounds are the same (*E*°′ = −0.219 V at pH = 7 and 30 °C in aqueous buffer) [59]. The estimated flavin occupancy for NQO2-F47 was 35% and for NQO2-L47 86%.

As expected, both variants were able to dimerise as shown by crosslinking with BS^3^ (Supplementary Fig. S2a). The addition of substrates and inhibitors had little effect on the crosslinking suggesting that they have limited effect on the overall conformation of the dimer (Supplementary Fig. S2b).

### 3.2 NQO2-F47 and NQO2-L47 have similar enzymatic activities in vitro

**Fig. 1:**
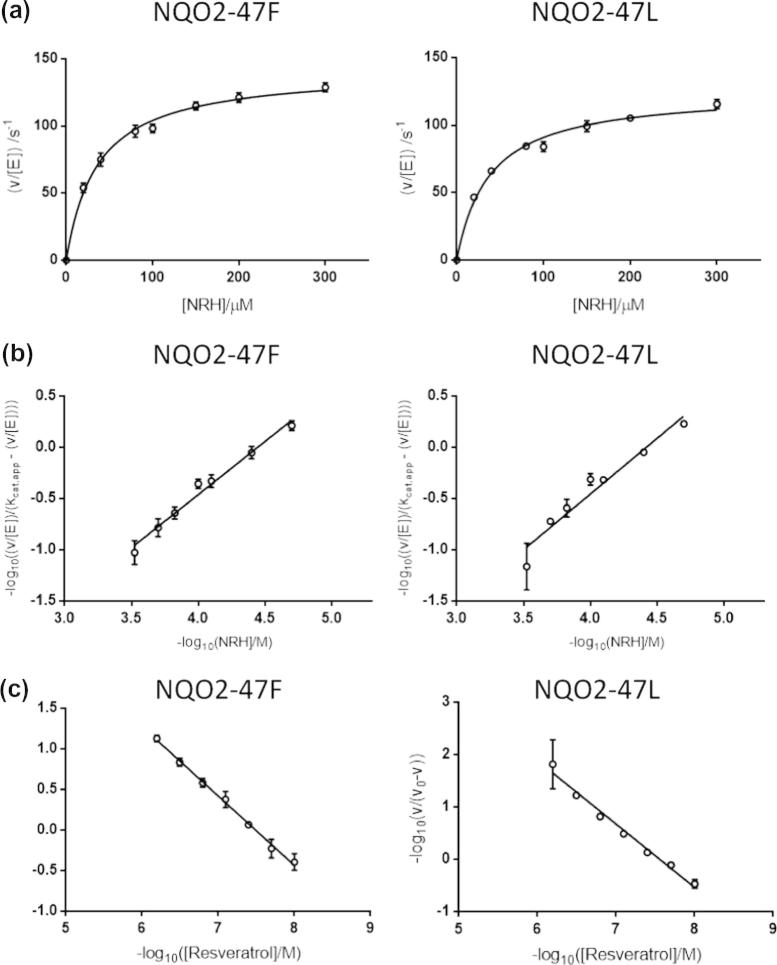
Steady-state enzyme kinetics of NQO2 variants. (a) The dependence of rate on NRH concentration was measured for both NQO2 variants (2.5 nM dimer) in the presence of 70 μM DCPIP. (b) Linear Hill plots were constructed from the data shown in (a) and used to estimate the Hill coefficient (*h*). (c) Linear Hill plots were constructed from inhibition data for both NQO2 variants (2.5 nM dimer) in the presence of 50 μM NRH, 70 μM DCPIP and varying amounts of resveratrol. In (a–c) each point represents the mean of three separate determinations and the error bar shows the standard error of these means.

Steady state kinetic analysis showed that the two variants have similar activities. The *K*_m,app_ values are similar; NQO-F47 has a specificity constant (*k*_cat_/*K*_m_) slightly higher than NQO-L47 (Fig. 1a; Table 1). This is consistent with reports that this variant has higher activity in cell extracts [48]. However, this small difference is unlikely to explain the majority of the observed variation. No cooperativity was observed with NRH as a substrate in either variant (Fig. 1b; Table 1). Both variants are inhibited by resveratrol, with similar apparent inhibition constants (Supplementary Fig. S3a; Table 1). Non-Michaelis-Menten kinetics have been observed with rat NQO1; this enzyme exhibits negative cooperativity towards the inhibitor dicumarol [60]. Interestingly, NQO2-F47 exhibits negative cooperativity towards resveratrol, but NQO2-L47 does not (Fig. 1c; Supplementary Fig. S3b; Table 1).

### 3.3 NQO2-F47 and NQO2-L47 have different stabilities towards proteolysis and thermal denaturation

**Fig. 2:**
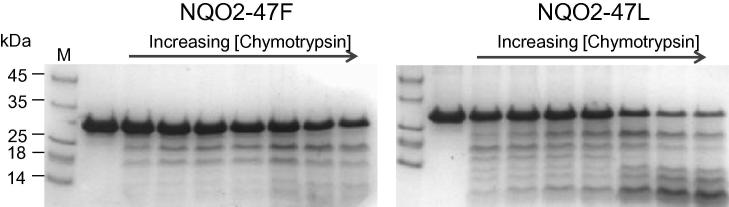
Limited proteolysis of the two common human NQO2 variants (35 μM) with chymotrypsin (0, 10, 35, 60, 90, 360, 630, 900 nM; 30 min at 37 °C) showed a greater effect on NQO2-L47 than NQO2-F47. The sizes of molecular mass markers (lane M) are shown to the left of the gel in kDa.

**Fig. 3:**
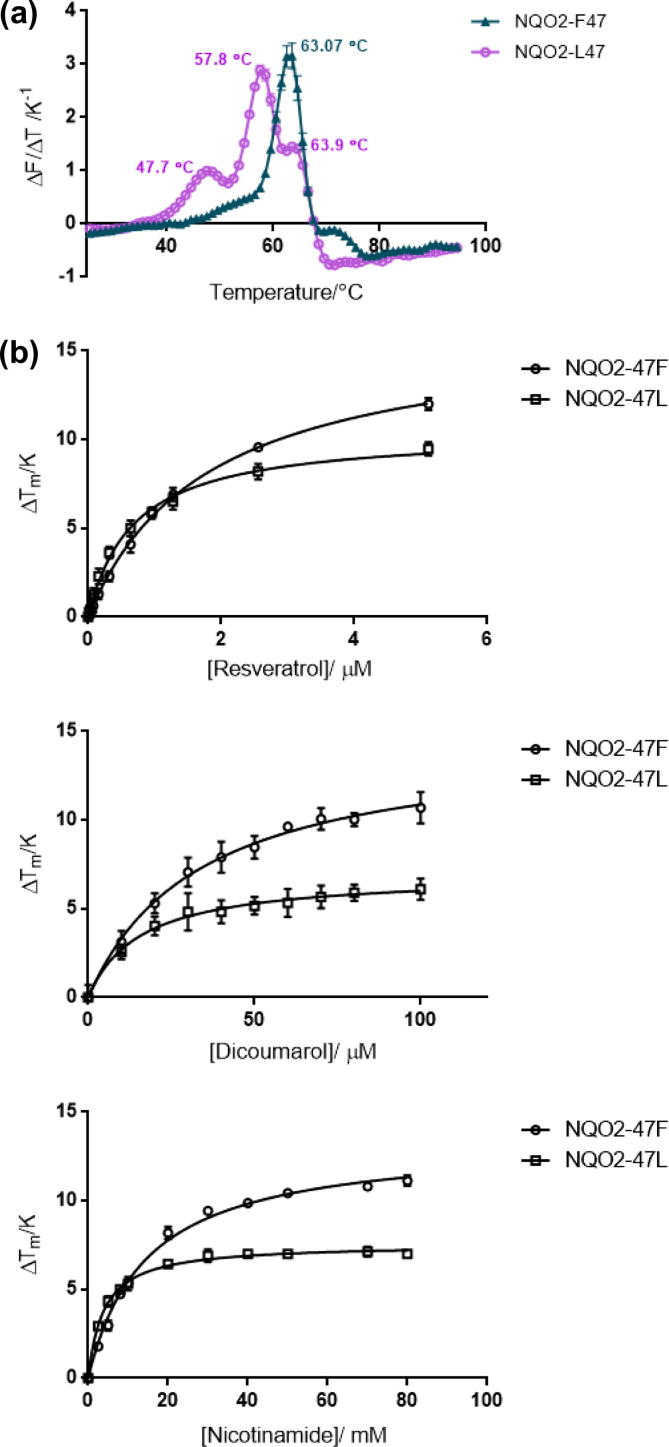
Thermal scanning fluorimetry of human NQO2 variants. (a) First derivative TSF scans for the thermal denaturation of both variants (0.5 μM). (b) Resveratrol, dicoumarol and nicotinamide have concentration-dependent effects on the melting temperature of both variants (0.5 μM).

NQO2-L47 was more susceptible to limited proteolysis by chymotrypsin than NQO2-F47 (Fig. 2). Similar results were also seen with trypsin and subtilisin (Supplementary Fig. S4). In TSF experiments, NQO2-L47 showed triphasic melting behaviour with transitions at 47.7 ± 0.3 °C, 57.8 ± 0.1 °C (major peak in the *dF*/*dT* plot) and 63.9 ± 0.3 °C. In contrast, NQO2-F47 displayed essentially monophasic behaviour with a single *T*_m_ of 63.1 ± 0.1 °C (Fig. 3a). This showed that NQO2-L47 exists in at least three different, metastable conformations in solution and that NQO2-F47 has slightly greater overall stability. (Note, the different peaks cannot arise from protein molecules with and without FAD bound since the assay measures the release of FAD from the protein; therefore, any NQO2 molecules lacking FAD are “invisible” to this assay.)

The significance of the multiple conformers observed in NQO2-L47 is unclear. Nevertheless, it is likely that the three conformers have slightly different properties, for example binding affinity for other proteins. This may be important when considering NQO2’s role as a signalling molecule since this requires interaction with proteins such as p53 [7]. There are precedents for this sort of behaviour. For example, mutations in the calcium sensor, calmodulin, result in altered interactions with the ion which alters the distribution of the protein’s conformational states and thus alters its affinities for protein binding partners [61].

Both variants are stabilised towards thermal denaturation by the addition of resveratrol, nicotinamide and dicoumarol in a concentration dependent manner (Fig. 3b). NQO2-F47 binds less tightly to these three ligands; the *K*_D,app_ values for resveratrol, nicotinamide and dicoumarol were 1.8 ± 0.1 μM, 14.4 ± 1.1 mM and 33.7 ± 2.6 μM, respectively. For NQO2-L47, these decrease to 0.7 ± 0.1 μM, 3.9 ± 0.2 mM and 15.7 ± 1.5 μM. The value obtained for resveratrol is higher than that obtained in previous studies (31 nM for NQO2-F47 by fluorescence quenching [34]). The concentration dependence of *T*_m_ in TSF assays is an indirect measure of the ligand’s affinity and so some variation might be expected. Nevertheless, studies on other proteins have established that the affinity rankings produced by TSF are consistent with those determined by isothermal titration calorimetry, for example [62,63]. As far as we can determine, no values for the affinity of NQO2-L47 for these ligands has been reported previously.

From these data, it is clear the lower activity of NQO2-L47 in cell extracts arises from several causes. A lower *k*_cat_/*K*_m_ makes a minor contribution. However, lower stability which, presumably, leads to reduced cellular concentrations of active enzyme is likely to be a more significant cause. Potentially, the higher affinity of NQO2-L47 for inhibitors could also contribute to its lower cellular activity since resveratrol is a component of the human diet. It is reasonable to assume that the different clinical outcomes associated with these NQO2 variants result from their different cellular activities. If so, then these ultimately result from the biochemical differences (especially protein stability) described here.

Increasing the concentration of ligand also altered the ratio of the fluorescence intensities at the three *T*_m_ values observed for NQO2-L47. The ratio of the intensities in the absence of added ligand (in order of increasing *T*_m_) was 0.1:1.0:1.2. In the presence of the highest concentration of resveratrol tested (5.12 μM) this changed to 0.1:1.0:0.4. That this ratio changes in response to ligand provides additional evidence for our hypothesis that there are at least three, interconverting, forms of NQO2-L47. At the same concentration of resveratrol, the thermal denaturation profile of NQO2-F47 remained essentially as one single transition (Supplementary Fig. S5).

Negative cooperativity widens the concentration range over which an enzyme responds to a substrate or inhibitor and requires communication between the active sites [64]. Residues 47’s location close to the dimer interface may be important in the transmission of information between the subunits in NQO2-F47. This communication between active sites requires protein flexibility [65]. Overall, NQO2-L47 is more flexible (based on proteolytic susceptibility and thermal denaturation), yet it is NQO2-F47 which exhibits negative cooperativity towards inhibitors. This suggests that the global flexibility observed in NQO2-L47 is not able to mediate cooperativity and that there must be some local flexibility in NQO2-F47. The lower flavin cofactor occupancy in NQO2-F47 may also contribute to the observed negative cooperativity. The physiological significance of this negative cooperativity is not currently known; similarly, the in vivo consequences of the lack of cooperativity in NQO2-L47 are not yet clear.

### 3.4 Conclusions

In inherited metabolic diseases, lower enzyme activity resulting from polymorphic forms of the protein is generally associated with the pathological state. Given the association between lower NQO2 activity and reduced cancer risk, the opposite may be true here. In which case, it is likely that resveratrol-mediated inhibition of NQO2 may be partly responsible for the health benefits claimed for this compound. However, the effects are likely to be complex since they will be affected by the multiple conformers of NQO2-L47 and the cooperativity of NQO2-F47.

## Figures and Tables

**Table 1:** Steady state enzyme kinetic parameters of NQO2 variants (2.5 nM) measured at 37 °C with NRH as the electron donor and DCPIP as the acceptor.

Parameter	NQO2-F47	NQO2-L47
*K*_m,app_(NRH)/μM^a^	36.1 ± 3.6	37.8 ± 3.4
*k*_cat,app_/s^−1^^a^	141 ± 4	125 ± 3
*k*_cat_/*K*_m_(NRH)/μM^−1^ s^a^^,^^b^	3.91 ± 0.61	3.31 ± 0.38
*h* (NRH as substrate)^a^	1.04 ± 0.07	1.09 ± 0.10
*K*_i,app_ (Resveratrol)/nM^c^	31.0 ± 4.3	16.7 ± 3.8
*h* (Resveratrol inhibition)^d^	0.85 ± 0.06	Not cooperative

a Measured with a constant DCPIP concentration of 70 μM and variable NRH concentrations.

b Note that, for a substituted enzyme mechanism, the specificity constant (*k*_cat_/*K*_m_) does not depend on the concentration of substrates.

c Measured with a constant DCPIP concentration of 70 μM, two different NRH concentrations and variable resveratrol concentrations; *K*_i,app_ values were estimated from a Dixon plot.

d Measured with a constant DCPIP concentration of 70 μM, constant NRH concentration of 50 μM and variable resveratrol concentrations. For NQO2-F47, the data fitted better to Eq. (3) compared to an equation lacking terms to account for cooperativity (*F* = 6.063, *P* < 0.01; see Section 2.3). For NQO2-L47, the data fitted better to a non-cooperative equation (*F* = 1.711; *P* > 0.2).

## Appendix A Supplementary data

Supplementary **figure S1** Expression and purification of human NQO2 varaints. Both NQO2-F47 and NQO2-L47 were expressed in, and purified from, *E. coli* as described in the Materials and Methods. The progress of the expression and purification was monitored by 10% SDS–PAGE. M, molecular mass markers (sizes shown to the left of the gel in kDa); Uninduced cells, cell extracts just prior to induction by IPTG; Induced cells, cell extracts 3 h after induction; Cell extract, material extracted from cells by sonication and clarified by centrifugation; Ni^2+^-flow, material which passed through the column; Ni^2+^-wash, material removed from the column by washing with wash buffer (50 mM HEPES-OH, pH 7.5, 500 mM sodium chloride, 10% glycerol); Elution, material removed from the column by washing with three 2 ml lots of elution buffer (wash buffer plus 250 mM imidazole). Proteins were then dialysed overnight (against dialysis buffer: 50 mM HEPES-OH, pH 7.5, 150 mM sodium chloride, 1 mM DTT, 10% glycerol) and stored frozen in 50–100 μl aliquots at −80 °C.

Supplementary **figure S2** Dimerisation of human NQO2 varaints. (a) Both NQO-F47 and NQOL47 (35 μM) can be crosslinked with BS^3^ (0, 50, 100, 200, 400, 800 μM; 30 min at 37 °C). BS^3^ was initially dissolved in 5 mM sodium citrate and the assays were performed in dialysis buffer (see Fig. S1). (b) The additions of potential NQO2 ligands did not greatly affect the pattern of crosslinking with BS^3^ (800 μM). Ligands were initially dissolved in 100% DMSO (resveratrol, curcumin), 0.13 M NaOH (dicoumarol) or 50 mM HEPES-OH, pH 7.5 (nicotinamide). NQO2 (35 μM) was tested alone and then in the presence of the final concentration of solvents used in the assay, i.e. 1% (v/v) DMSO (1.0%) and 1.3 mM NaOH (1.0% v/v of 0.13 M NaOH). Ligands were tested at the following final concentrations: Resveratrol (0.5 μM), Dicoumarol (200 μM), Curcumin (40 μM) and Nicotinamide (160 mM). Similar results were observed with the crosslinker EDC (64 mM; stock solutions dissolved in water) under similar conditions (data not shown).

Supplementary **figure S3** Inhibition of NQO2 variants by resveratrol. (a) Dixon plots showing the inhibition of NQO2-F47 and NQO2-L47 (2.5 nM) by resveratrol with DCPIP (70 μM) and two different concentrations of NRH (50 and 100 μM). These plots were used to estimate *K*_i,app_. (b) Non-linear fits to Eq. (3) which were used to determine whether, or not, the inhibition was cooperative and, if so, the value of the Hill coefficient (*h*) – see Section 2.3. The rates of DCPIP (70 μM) reduction by NRH (50 μM) in the presence of a range of concentrations of resveratrol was measured with both variants (2.5 nM). In both (a) and (b), each point represents the mean of three determinations (same enzyme preparation, same diluted stock) and the error bars the standard errors of these means.

Supplementary **figure S4** Limited proteolysis of human NQO2 variants. (a) Limited proteolysis of the two NQO2 variants (35 μM) with trypsin (0, 10, 35, 60, 90, 360, 630, 900 nM; 30 min at 37 °C) showed a greater effect on NQO2-L47 than NQO2-F47. (b) Limited proteolysis of the two NQO2 variants (35 μM) with subtilisin (0, 10, 35, 60, 90, 360, 630, 900 nM; 30 min at 37 °C) showed a greater effect on NQO2-L47 than NQO2-F47. In both (a) and (b), the sizes of molecular mass markers (lane M) are shown to the left of the gel in kDa.

Supplementary **figure S5** Thermal scanning fluorimetry of human NQO2 variants in the presence of resveratrol. First derivative TSF scans for the thermal denaturation of both variants (0.5 μM) in the presence of resveratrol (5.12 μM).
