# Supplementary material for: The two common polymorphic forms of human NRH-quinone oxidoreductase 2 (NQO2) have different biochemical properties
Source: FEBS Lett. 2014 May 2;588(9):1666–72. doi: 10.1016/j.febslet.2014.02.063 (PMC4045209; doi:10.1016/j.febslet.2014.02.063)
Supplement: Supplementary figure S1 — Expression and purification of human NQO2 varaints. Both NQO2-F47 and NQO2-L47 were expressed in, and purified from, E. coli as described in the Materials and Methods. The progress of the expression and purification was monitored by 10% SDS–PAGE. M, molecular mass markers (sizes shown to the left of the gel in kDa); Uninduced cells, cell extracts just prior to induction by IPTG; Induced cells, cell extracts 3 h after induction; Cell extract, material extracted from cells by sonication and clarified by centrifugation; Ni2+-flow, material which passed through the column; Ni2+-wash, material removed from the column by washing with wash buffer (50 mM HEPES-OH, pH 7.5, 500 mM sodium chloride, 10% glycerol); Elution, material removed from the column by washing with three 2 ml lots of elution buffer (wash buffer plus 250 mM imidazole). Proteins were then dialysed overnight (against dialysis buffer: 50 mM HEPES-OH, pH 7.5, 150 mM sodium chloride, 1 mM DTT, 10% glycerol) and stored frozen in 50–100 μl aliquots at −80 °C. [file mmc1.pptx]

## Slide 1
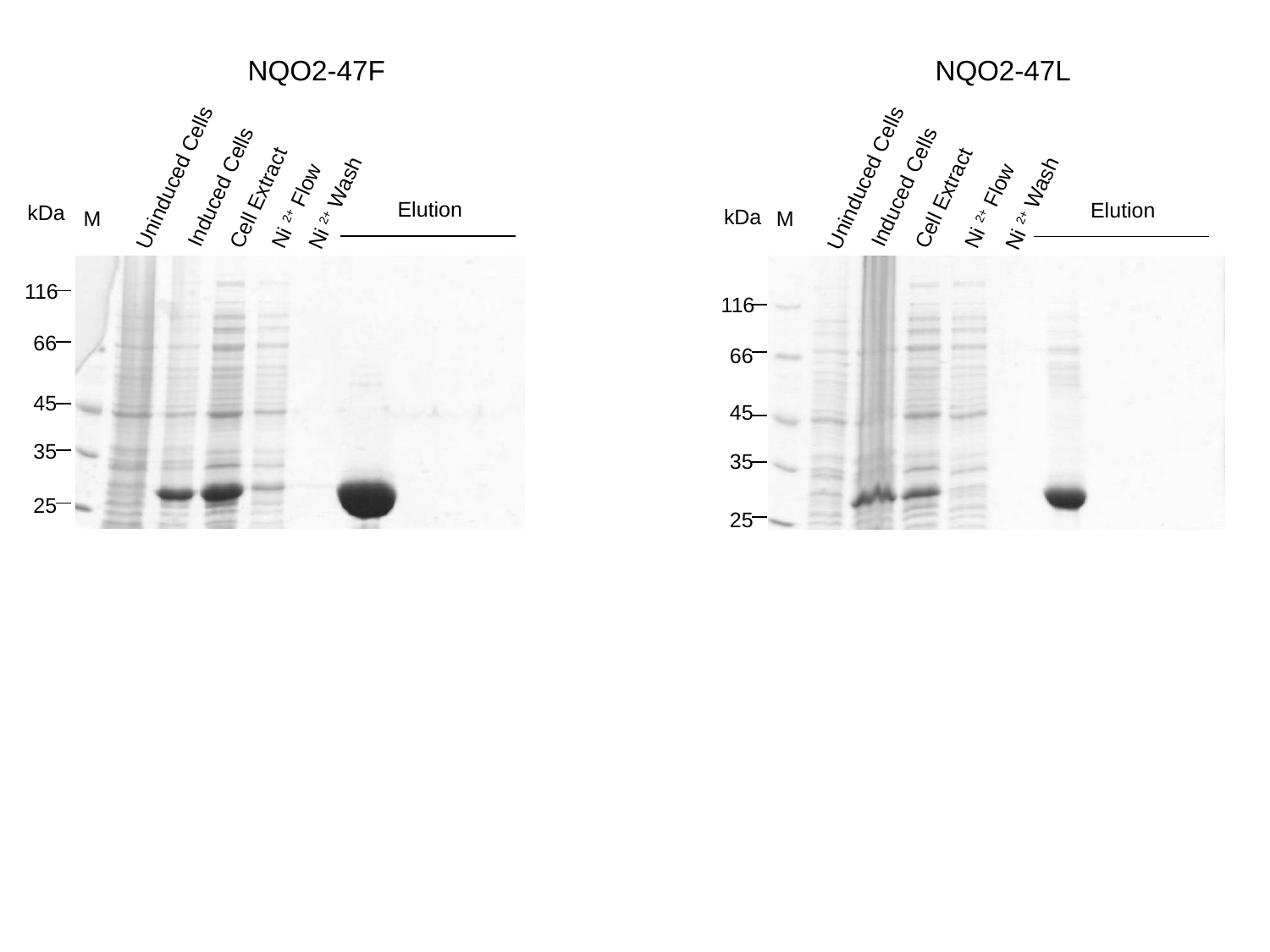

NQO2-47F
NQO2-47L
Uninduced Cells
Induced Cells
Cell Extract
Ni 2+ Wash
Ni 2+ Flow
Elution
M
kDa
116
66
45
35
25
Uninduced Cells
Induced Cells
Cell Extract
Ni 2+ Wash
Ni 2+ Flow
Elution
M
kDa
116
66
45
35
25
