# Supplementary material for: The two common polymorphic forms of human NRH-quinone oxidoreductase 2 (NQO2) have different biochemical properties
Source: FEBS Lett. 2014 May 2;588(9):1666–72. doi: 10.1016/j.febslet.2014.02.063 (PMC4045209; doi:10.1016/j.febslet.2014.02.063)
Supplement: Supplementary figure S2 — Dimerisation of human NQO2 varaints. (a) Both NQO-F47 and NQOL47 (35 μM) can be crosslinked with BS3 (0, 50, 100, 200, 400, 800 μM; 30 min at 37 °C). BS3 was initially dissolved in 5 mM sodium citrate and the assays were performed in dialysis buffer (see Fig. S1). (b) The additions of potential NQO2 ligands did not greatly affect the pattern of crosslinking with BS3 (800 μM). Ligands were initially dissolved in 100% DMSO (resveratrol, curcumin), 0.13 M NaOH (dicoumarol) or 50 mM HEPES-OH, pH 7.5 (nicotinamide). NQO2 (35 μM) was tested alone and then in the presence of the final concentration of solvents used in the assay, i.e. 1% (v/v) DMSO (1.0%) and 1.3 mM NaOH (1.0% v/v of 0.13 M NaOH). Ligands were tested at the following final concentrations: Resveratrol (0.5 μM), Dicoumarol (200 μM), Curcumin (40 μM) and Nicotinamide (160 mM). Similar results were observed with the crosslinker EDC (64 mM; stock solutions dissolved in water) under similar conditions (data not shown). [file mmc2.pptx]

## Slide 1
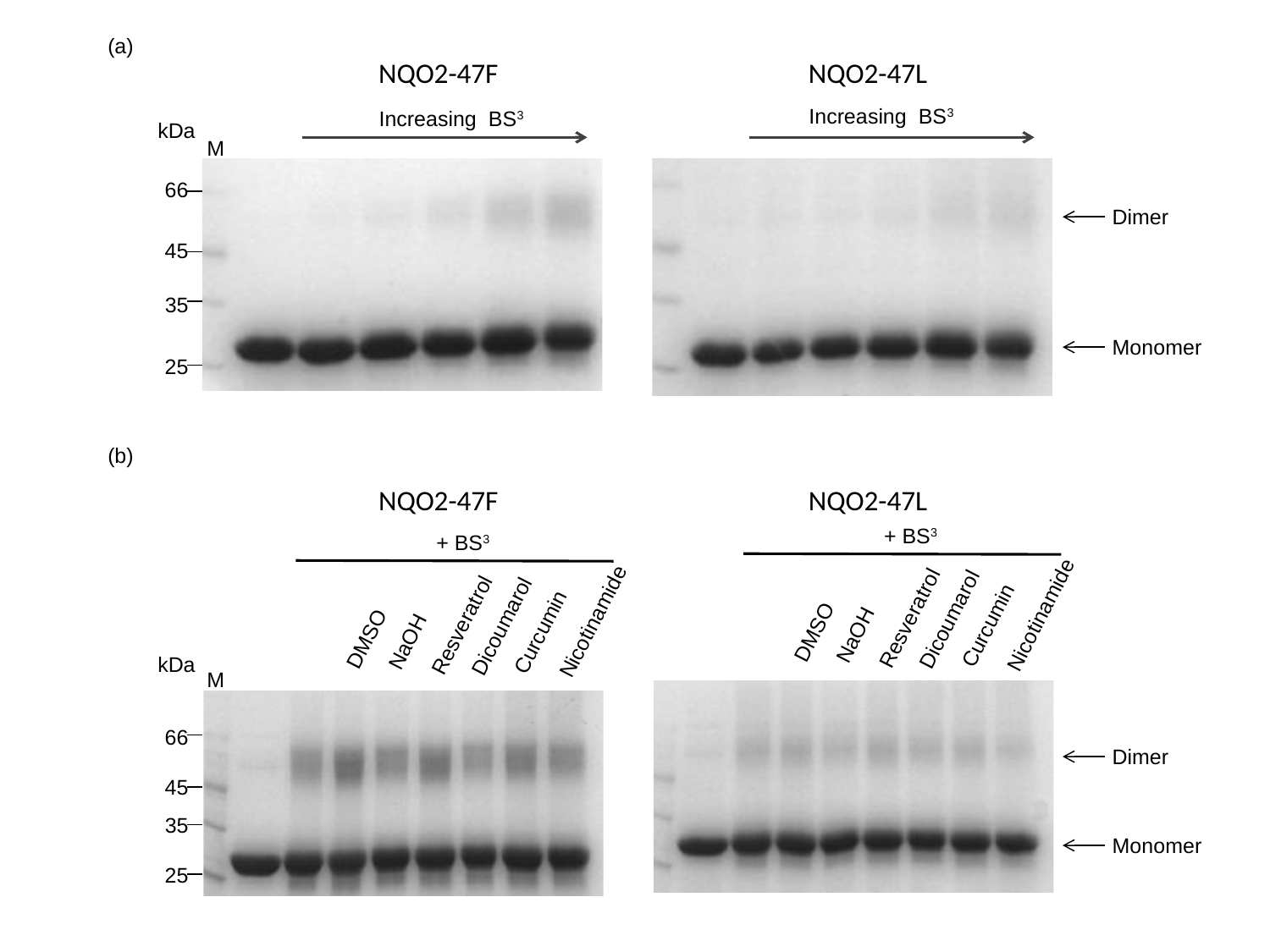

(a)
NQO2-47F
NQO2-47L
Increasing BS3
Increasing BS3
kDa
M
66
45
35
25
Dimer
Monomer
(b)
NQO2-47F
NQO2-47L
+ BS3
Nicotinamide
Resveratrol
Dicoumarol
Curcumin
DMSO
NaOH
+ BS3
Nicotinamide
Resveratrol
Dicoumarol
Curcumin
DMSO
NaOH
kDa
M
66
45
35
25
Dimer
Monomer
