# Supplementary figures and images for: The two common polymorphic forms of human NRH-quinone oxidoreductase 2 (NQO2) have different biochemical properties
Source: FEBS Lett. 2014 May 2;588(9):1666–72. doi: 10.1016/j.febslet.2014.02.063 (PMC4045209; doi:10.1016/j.febslet.2014.02.063)

## Slide 1
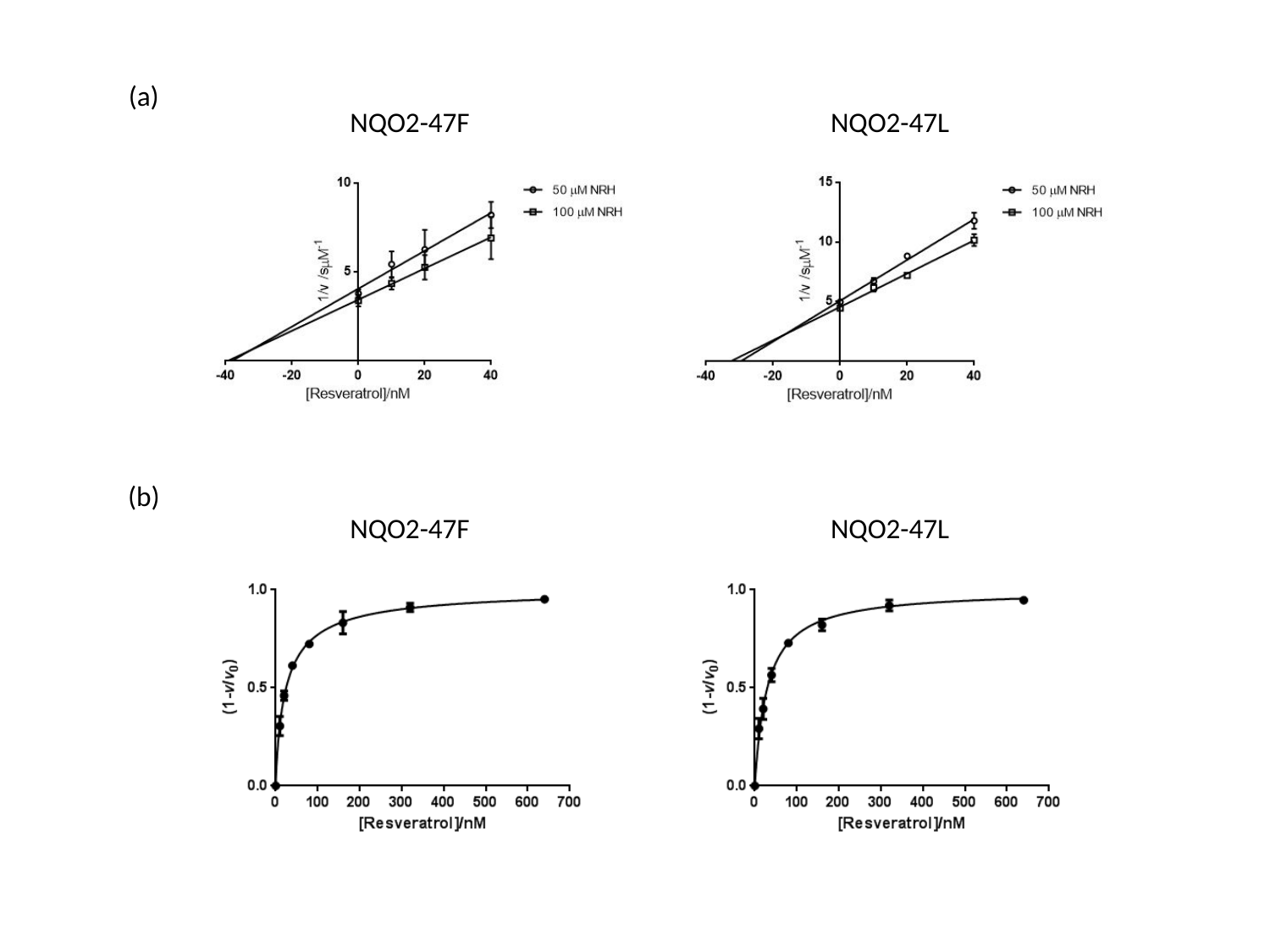

(a)
NQO2-47F
NQO2-47L
(b)
NQO2-47F
NQO2-47L

Supplement: Supplementary figure S3 — Inhibition of NQO2 variants by resveratrol. (a) Dixon plots showing the inhibition of NQO2-F47 and NQO2-L47 (2.5 nM) by resveratrol with DCPIP (70 μM) and two different concentrations of NRH (50 and 100 μM). These plots were used to estimate Ki,app. (b) Non-linear fits to Eq. (3) which were used to determine whether, or not, the inhibition was cooperative and, if so, the value of the Hill coefficient (h) – see Section 2.3. The rates of DCPIP (70 μM) reduction by NRH (50 μM) in the presence of a range of concentrations of resveratrol was measured with both variants (2.5 nM). In both (a) and (b), each point represents the mean of three determinations (same enzyme preparation, same diluted stock) and the error bars the standard errors of these means. [file mmc3.pptx]

## Slide 1
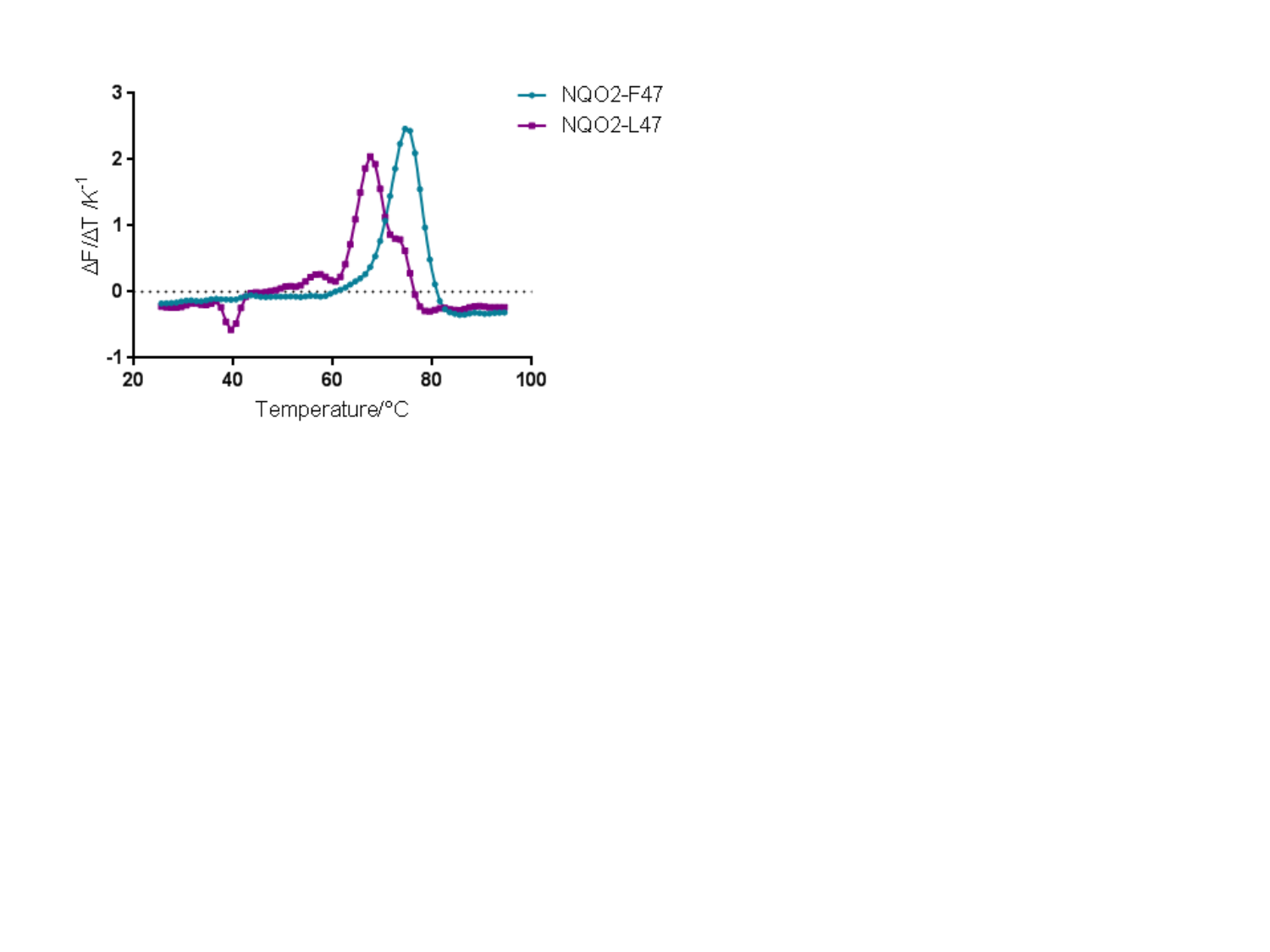

Supplement: Supplementary figure S5 — Thermal scanning fluorimetry of human NQO2 variants in the presence of resveratrol. First derivative TSF scans for the thermal denaturation of both variants (0.5 μM) in the presence of resveratrol (5.12 μM). [file mmc5.pptx]
