# Supplementary material for: The two common polymorphic forms of human NRH-quinone oxidoreductase 2 (NQO2) have different biochemical properties
Source: FEBS Lett. 2014 May 2;588(9):1666–72. doi: 10.1016/j.febslet.2014.02.063 (PMC4045209; doi:10.1016/j.febslet.2014.02.063)
Supplement: Supplementary figure S4 — Limited proteolysis of human NQO2 variants. (a) Limited proteolysis of the two NQO2 variants (35 μM) with trypsin (0, 10, 35, 60, 90, 360, 630, 900 nM; 30 min at 37 °C) showed a greater effect on NQO2-L47 than NQO2-F47. (b) Limited proteolysis of the two NQO2 variants (35 μM) with subtilisin (0, 10, 35, 60, 90, 360, 630, 900 nM; 30 min at 37 °C) showed a greater effect on NQO2-L47 than NQO2-F47. In both (a) and (b), the sizes of molecular mass markers (lane M) are shown to the left of the gel in kDa. [file mmc4.pptx]

## Slide 1
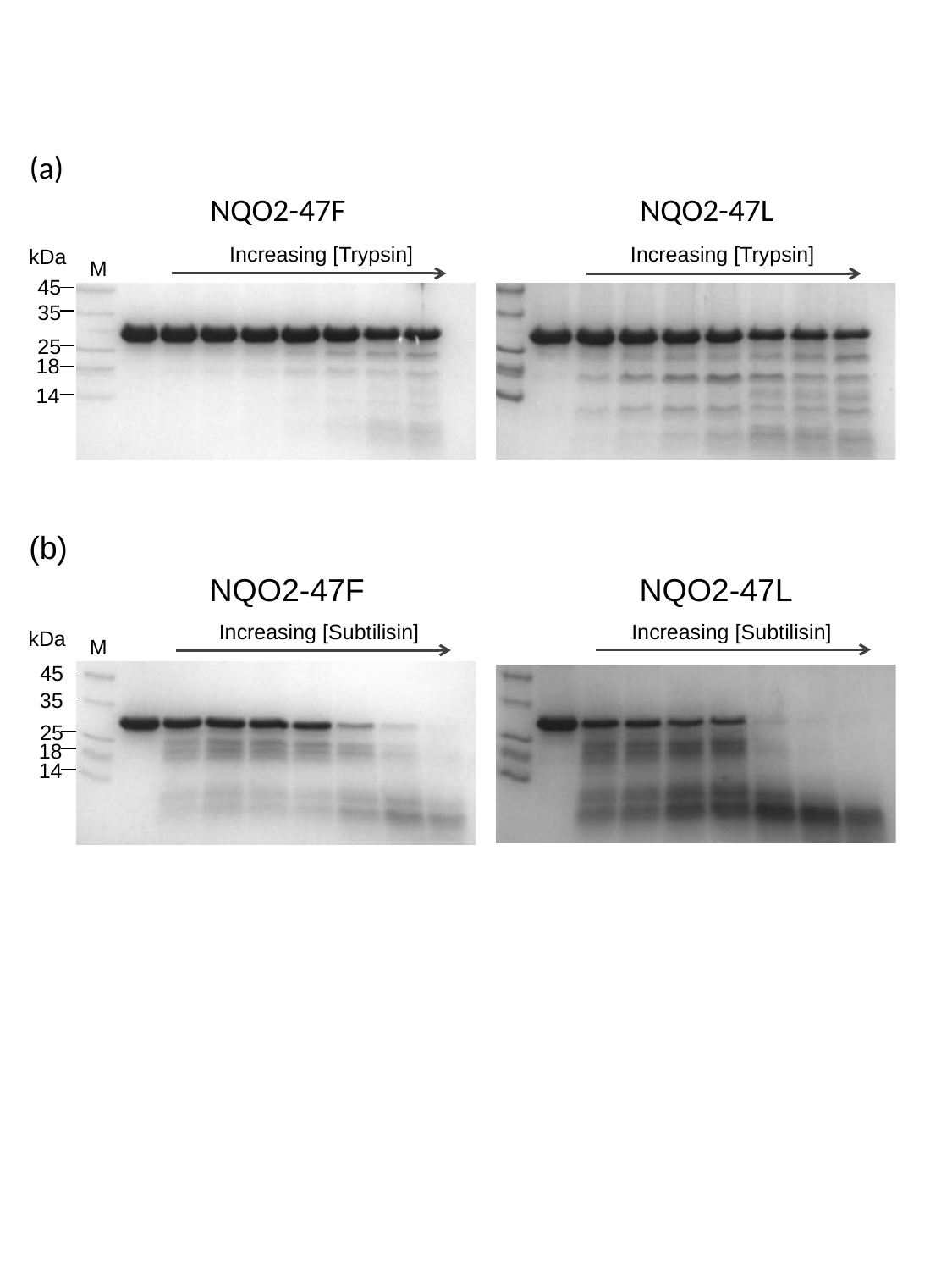

(a)
NQO2-47F
NQO2-47L
Increasing [Trypsin]
kDa
M
45
35
25
18
14
Increasing [Trypsin]
(b)
NQO2-47F
NQO2-47L
Increasing [Subtilisin]
kDa
M
45
35
25
18
14
Increasing [Subtilisin]
